# Genetic Predisposition to Prediabetes in the Kazakh Population

**DOI:** 10.3390/cimb46100648

**Published:** 2024-09-28

**Authors:** Gulnara Svyatova, Galina Berezina, Alexandra Murtazaliyeva, Altay Dyussupov, Tatyana Belyayeva, Raida Faizova, Azhar Dyussupova

**Affiliations:** 1 Laboratory of Republican Medical Genetic Consultation, Scientific Center of Obstetrics, Gynecology, and Perinatology, Almaty 050020, Kazakhstan; gsvyatova1@mail.ru (G.S.); gberezina54@mail.ru (G.B.); alexmurtazalieva@gmail.com (A.M.); 2 Department of General Medical Practice, Semey Medical University, Semey 071400, Kazakhstan; altay.dyusupov@smu.edu.kz (A.D.); btm56@rambler.ru (T.B.); pochtaamir@mail.ru (R.F.)

**Keywords:** prediabetes, gene polymorphisms, GWAS analysis, minor alleles

## Abstract

The aim of this study was to conduct a comparative analysis of the population frequencies of the minor allele of polymorphic variants in the genes *TCF7L2* (rs7903146) and *PPARG* (rs1801282), based on the genome-wide association studies analysis data associated with the risk of developing prediabetes, in an ethnically homogeneous Kazakh population compared to previously studied populations worldwide. This study utilized a genomic database consisting of 1800 ethnically Kazakh individuals who were considered in healthy condition. Whole-genome genotyping was performed using Illumina OmniChip 2.5–8 arrays, which interrogated approximately 2.5 million single nucleotide polymorphisms. The distribution of genotypes for the *TCF7L2* (rs7903146) and *PPARG* (rs1801282) polymorphisms in the Kazakh sample was found to be in Hardy–Weinberg equilibrium (*p* > 0.05). The minor G allele of the “Asian” protective polymorphism rs1801282 in the *PPARG* gene was observed at a frequency of 13.8% in the Kazakh population. This suggests a potentially more significant protective effect of this polymorphism in reducing the risk of prediabetes among Kazakhs. The frequency of the unfavorable T allele of the insulin secretion-disrupting gene *TCF7L2* (rs7903146) in Kazakhs was 15.2%. Studying the associations of genetic markers for prediabetes enables the timely identification of “high-risk groups” and facilitates the implementation of effective preventive measures. Further results from replicative genomic research will help identify significant polymorphic variants of genes underlying the alteration of prediabetes status.

## 1. Introduction

Prediabetes is a transitional state between normal glucose regulation and type 2 diabetes (T2D), where insulin resistance (impaired glucose uptake by tissues) has not yet progressed to the stage of clinical disease. The etiology of prediabetes is still not fully understood, although it is believed that disturbances in carbohydrate metabolism and obesity play significant roles.

Type 2 diabetes is a complex disorder influenced by genetic and environmental factors. It is characterized by chronic hyperglycemia resulting from an insufficient response to insulin and is currently the most prevalent metabolic disorder worldwide, affecting over 463 million individuals [1,2]. Risk factors contributing to the increased incidence of T2D include excess body weight, lack of physical activity, poor dietary habits, genetics, family history of diabetes, and older age.

The global obesity epidemic has led to a rapid increase in the prevalence of cardiometabolic disorders, including type 2 diabetes [3,4]. It is well established that obesity, prediabetes, and insulin resistance are closely interconnected [5,6,7,8]. While prediabetes is an inevitable stage for individuals progressing to T2D, it is important to note that not all individuals with prediabetes will ultimately develop diabetes. The annual conversion rate from prediabetes to diabetes is estimated to be around 5–10% [9].

To date, the precise pathogenesis of T2D remains incompletely understood. However, whole-genome association studies have already identified over 100 genetic loci that are significantly associated with increased susceptibility to T2D, confirming the crucial role of inherited factors in its onset and development [1,3,4,10]. Consequently, substantial efforts have been made to identify genes related to T2D, and many loci associated with the disease were discovered through genetic association studies and genome-wide association studies (GWAS) [5,6,7,8,11,12]. None of these studies, however, have investigated changes in prediabetic status.

Prediabetes is recognized as a distinct state between health and diabetes, characterized by elevated blood glucose levels that do not meet the diagnostic criteria for diabetes [13]. Impaired glucose tolerance (IGT) and impaired fasting glucose (IFG) are considered high-risk factors for prediabetes and are defined by fasting plasma glucose (FPG), 2 h postprandial glucose (2 h PG), and glycated hemoglobin (HbA1C) levels [14,15].

The World Health Organization (WHO) [16] does not recommend using the terms “prediabetes” and “borderline diabetes”, as a high risk does not necessarily imply an inevitable diabetes diagnosis. The International Diabetes Federation in 2021 [17] projected that the global prevalence of prediabetes could reach 531.6 million individuals by 2045. There are no universally agreed-upon diagnostic criteria for prediabetes. The WHO [16] is currently discussing the possibility of using HbA1c for its diagnosis, while the American Diabetes Association (ADA) [18] has approved different levels of HbA1c for diagnosing prediabetes. Nevertheless, international diabetes associations concur that IGT following a 2 h oral glucose tolerance test remains the primary criterion for a prediabetes diagnosis.

The World Health Organization (WHO) defined prediabetes as a state of intermediate hyperglycemia using two specific parameters: impaired fasting glucose (IFG), defined as fasting plasma glucose (FPG) levels of 6.1–6.9 mmol/L (110–125 mg/dL), and impaired glucose tolerance (IGT), defined as 2 h plasma glucose levels of 7.8–11.0 mmol/L (140–200 mg/dL) following the ingestion of 75 g of oral glucose or its combination based on a 2 h oral glucose tolerance test (OGTT) [16].

One of the main risk factors for developing prediabetes is obesity, which has become a global problem and accounts for 80–85% of all cases of this condition. It has been shown that lifestyle modification in prediabetes, such as increasing physical activity (from 2.5 to 4 h per week), dietary changes, as well as weight reduction, improves blood glucose control and reduces the risk of developing diabetes by more than 50% [19,20]. Other common risk factors include lifestyle factors, environmental conditions, social status, family history, presence of relatives with T2D, and ethnic background [21]. For example, T2D occurs almost six times more frequently in individuals of South Asian origin and three times more frequently in African Americans, with an onset approximately 10 years earlier than in Europeans [22].

The main clinical problem of prediabetes is the high risk of progressing to T2D. However, prediabetes itself is associated with a range of negative health consequences [9], including chronic kidney disease, nephropathy, neuropathy, retinopathy, and macrovascular diseases [23]. A meta-analysis of 760,925 cases of prediabetes found a 21% increased risk of stroke [24].

Thus, despite some differences in diagnostic criteria, international diabetes associations unanimously assert that prediabetes is a borderline state of T2D and poses a significant health risk to the population, increasing morbidity and mortality. Research focusing on studying the genetic contribution of significant T2D genomic loci to the risk of developing and progressing prediabetes in different ethnic populations is recommended, taking into account environmental factors, lifestyle, and family history [25]. Such studies will help identify new genes for prognostic markers and the development of effective preventive measures for T2D.

The aim of this study was to assess the frequencies of two minor alleles of polymorphic variants in the *TCF7L2* gene (rs7903146) and the *PPARG* gene (rs1801282), which are associated with the risk of prediabetes, in an ethnically homogeneous population of Kazakhs.

## 2. Materials and Methods

### 2.1. Study Setting and Patient Enrollment

The genomic information of 1800 apparently healthy individuals of Kazakh nationality was used as the study material. A sample size of 1800 allows us to have a sufficient number of individuals carrying the minor alleles, which is crucial for detecting associations with moderate effect sizes. With an MAF of 0.1378 for *PPARG* (rs1801282) and 0.1521 for *TCF7L2* (rs7903146), we expected approximately 500 carriers of each risk allele, providing adequate variation for meaningful analysis. We conducted a power analysis based on the disease prevalence of 3.7% in the Kazakh population and common effect sizes reported in the literature for these polymorphisms. Specifically, for *TCF7L2* (rs7903146), odds ratios (OR) between 1.4 and 2.0 were observed, while for *PPARG* (rs1801282), a protective effect with ORs around 0.8 is typical. With a significance level of 0.05, our sample of 1800 provides over 80% power to detect moderate-to-large effect sizes for both polymorphisms, ensuring that our study was well powered to identify genetic associations.

The participants were informed about the objectives of the project and provided informed consent. The study design was cross-sectional.

The criteria for the population control group: ethnicity—Kazakhs up to the third generation; legal capacity. The exclusion criteria: history of obesity, type 1 or type 2 diabetes confirmed by medical documentation, history of prediabetes according to WHO criteria [16].

DNA samples from the population control group were stored in the “Miras” Biobank (Scientific Center of Obstetrics, Gynecology, and Perinatology, Almaty, Kazakhstan), which was established as part of the InterPregGen project under Grant Agreement No. 282540 of the 7th Framework Programme of the European Commission.

To select polymorphisms associated with the development of prediabetes, global databases were utilized: ENCODE (http://genome.ucsc.edu/encode/, accessed on 15 April 2024), summary statistics from the Roadmap Epigenomics Project (http://genomebrowser.wustl.edu/, accessed on 15 April 2024), HaploRegV4 (http://compbio.mit.edu/HaploReg, accessed on 15 April 2024), (www.ebi.ac.uk/gwas, accessed on 15 April 2024), major consortia (MAGIC and GWAMA DIAGRAM), genotype and phenotype databases (www.ncbi.nlm.nih.gov/gap, accessed on 15 April 2024), National Human Genome Research Institute (NHGRI) Catalog of Genome-Wide Association Studies (www.genome.gov, accessed on 15 April 2024), HapMap project (http://hapmap.ncbi.nlm.nih.gov/index.html.en, accessed on 15 April 2024), and Ensembl genome database project (http://asia.ensembl.org/index.html, accessed on 15 April 2024).

Two polymorphic variants, *PPARG* (Peroxisome proliferator-activated receptor gamma, rs1801282), and *TCF7L2* (Transcription Factor 7 Like 2, rs7903146), were selected for studying the population characteristics associated with prediabetes. Table 1 presents the genetic characteristics of the investigated gene polymorphisms, *PPARG* (rs1801282) and *TCF7L2* (rs7903146), which, according to the results of conducted GWAS in various ethnic populations, demonstrated significant associations with the risk of developing type 2 diabetes.

### 2.2. Methods of This Study

DNA extraction was performed using an automated analyzer, Prepitto (PerkinElmer, Waltham, MA, USA), utilizing the magnetic particle separation method with M-PVA beads. Genotyping was conducted using Illumina OmniChip 2.5–8 arrays at the DECODE Iceland Genomic Center as part of the InterPregGen project. Quality control for genotyping was carried out, excluding SNPs with a minimal allele frequency (MAF) below 1%, call rate less than 98%, significance level less than *p* < 0.05, and deviation from Hardy–Weinberg equilibrium (*p* < 0.05) [25].

### 2.3. Ethics Statement

This study was approved by the local ethics committee of the Nonprofit Joint-Stock Company “Semey Medical University” (Semey, Kazakhstan), protocol number 6 (G-041.11.01.03-202r) dated 22 February 2022 and by the Bioethics Committee of Scientific Center of Obstetrics, Gynecology, and Perinatology, Ministry of Health and Social Development of the Republic of Kazakhstan (Almaty, Kazakhstan), protocol number 2 dated 28 June 2016.

### 2.4. Statistical Analysis 

Statistical processing was performed using PLINK software v1.90. The HWE test function in PLINK was employed to assess the concordance of genotype frequencies with Hardy–Weinberg equilibrium [26]. The significance in all statistical procedures was considered at *p* < 0.05 [26,27].

## 3. Results

Population characteristics of allelic and genotypic distributions of the *PPARG* (Peroxisome proliferator-activated receptor gamma, rs1801282) and *TCF7L2* (Transcription Factor 7 Like 2, rs7903146) gene polymorphisms, potentially associated with the development of prediabetes, were examined based on genotyping data from 1800 individuals of Kazakh ethnicity.

Table 2 displays the minor allele frequencies of the gene polymorphisms, *PPARG* (rs1801282) and *TCF7L2* (rs7903146), associated with prediabetes, in the studied Kazakh population. The population frequency of the minor allele for the rs1801282 polymorphism of the *PPARG* gene was 13.78%, while a higher population frequency MAF was observed for the rs7903146 polymorphism of the *TCF7L2* gene, reaching 15.21% in the studied Kazakh population. The genotype distribution for the studied polymorphisms in the Kazakh population is in accordance with Hardy–Weinberg equilibrium, as the differences between the expected and observed heterozygosity for both polymorphisms were not significant (*p* > 0.05).

Table 3 provides the results of comparative characteristics of allele frequencies of the *PPARG* (rs1801282) and *TCF7L2* (rs7903146) gene polymorphisms in the Kazakh population and global populations. Notably, no studies investigating these polymorphisms in relation to prediabetes have been conducted. We also found an absence of significant differences in the frequency of carrying the minor G allele in the Kazakh population compared to European populations such as England (12.1%), Spain (11.7%), and Italy (8.4%) (*p* > 0.05), for which a protective role of the rs1801282 polymorphism in the *PPARG* gene has been described, suggesting a similar protective effect for Kazakhs. It is noteworthy that the population frequency of the G allele of this polymorphism in East Asian populations, such as China (4.9%), Japan (2.9%), and Vietnam (1.0%), was significantly lower than that of Kazakhs (*p* < 0.05), but did not differ from its frequency in South Asian populations, Bangladesh (11.0%), India (9.2%), and Pakistan (14.1%) (*p* > 0.05). Our study indicates that the population frequency of carrying the unfavorable minor G allele of the rs1801282 polymorphism in the *PPARG* gene among the surveyed 1800 Kazakh individuals was 13.8%, which did not significantly differ from the populations of Europe (12.0%) and South Asia (12.0%) (*p* > 0.05) but was significantly higher than that of the East Asian population (2.6%) (*p* < 0.001). The frequency of the minor T allele of the rs7903146 polymorphism in the *TCF7L2* gene in the Kazakh study group was 15.2%, which is significantly higher than the corresponding frequencies in the East Asian population (2.3%) (*p* < 0.001), but significantly lower than its frequencies in European (31.7%) and South Asian (29.9%) populations (*p* < 0.001). The comparative analysis with specific populations from Europe, East Asia, and South Asia presented in Table 3 confirms that the population frequency of the minor T allele of the rs7903146 polymorphism in the *TCF7L2* gene in the Kazakh population occupies an intermediate position between the previously studied populations of Europe and South Asia and the population of East Asia (*p* < 0.001). 

## 4. Discussion

The high population frequency of carrying the minor G allele of the rs180128 polymorphism in the *PPARG* gene in the studied Kazakh sample, the conflicting results, and the significant ethnic differences observed, along with the high geographic diversity of its population frequencies, justified our choice of this polymorphism for investigating its potential role as a protective marker for the development of prediabetes in the Kazakh population.

The peroxisome proliferator-activated receptor gamma (*PPARG*) gene encodes the gamma receptor activated by peroxisome proliferators [28]. *PPARG* serves as a regulator of adipocyte and fatty acid differentiation [29] and influences glucose metabolism. Several GWAS have identified a protective effect of the minor G allele of the rs1801282 polymorphism in the PPARG gene, which regulates carbohydrate and lipid metabolism, with a decreased risk of type 2 diabetes (T2D) observed in Asian populations but not in European populations [30,31]. Significant ethnic differentiation in the population frequencies of the rs1801281 polymorphism in the *PPARG* gene was reported by many researchers, for instance, 12% in European populations, 10% in Native Americans, 4% in the Japanese population, 3% in African Americans, and 1% in Chinese individuals [32]. It was demonstrated that the rs1801282 polymorphism in the *PPARG* gene is associated with a lower body mass index (BMI), increased insulin sensitivity, and a reduced risk of T2D [33,34], indicating a potential protective effect of the minor G allele, at least in European populations [35]. However, the genetic contribution of the rs1801282 polymorphism in the development of prediabetes has not been previously investigated, which motivated its selection for replication studies in the Kazakh population. A GWAS study or meta-analysis on prediabetes was conducted specifically for this polymorphism. However, a meta-analysis of 2858 patients with gestational diabetes mellitus (GDM) and 6890 controls from nine published case–control studies demonstrated a protective effect of carrying the minor G allele of the rs180128 polymorphism in the *PPARG* gene (OR = 0.89, 95% CI: 0.77–1.04, *p* = 0.015). As we previously reported in an article [36], these results contradict the meta-analysis conducted by Du, J., et al. (2012), which showed a significant association of the rs1801282 polymorphism in the *PPARG* gene with a reduced risk of GDM only in Asian populations (four studies, 1197 GDM cases compared to 1026 controls; OR = 0.72, 95% CI: 0.56–0.93), but not in Europeans (six studies, 1732 GDM cases compared to 5943 controls; OR = 1.07, 95% CI: 0.91–1.18) [37].

It is known that the Transcription Factor 7 Like 2 (*TCF7L2*) gene encodes T-cell transcription factor 4, a transcription factor of the Wnt/β-catenin signaling pathway that is crucial for pancreatic islet embryogenesis and regulation of blood glucose levels. Several genome-wide association studies showed that the *TCF7L2* (rs7903146) polymorphism can significantly influence individual susceptibility to type 2 diabetes (T2D) in specific populations [38,39,40]. The results of large-scale association studies by Ding W. et al. (2018) involving 34,232 T2D patients and 22,396 controls revealed a significant genetic contribution of the rs7903146 polymorphism in the *TCF7L2* gene to the development of T2D. The association was observed using a dominant model (OR = 1.41, 95% CI: 1.36–1.47, *p* < 0.0001), recessive model (OR = 1.58, 95% CI: 1.48–1.69, *p* < 0.0001), additive model (OR = 1.34, 95% CI: 1.28–1.39, *p* < 0.0001), and allele model (OR = 1.35, 95% CI: 1.31–1.39, *p* < 0.0001) in individuals of European, East Asian, South Asian, and other ethnicities [41]. Similar results were obtained in an extended meta-analysis conducted by Lou, L., et al. (2019) involving 68 studies with 115,809 participants with type 2 diabetes (T2D). The analysis revealed a substantial association between the *TCF7L2* rs7903146 polymorphism and a predisposition to T2D in Asian and European populations across all genetic models examined (dominant, recessive, allele) (*p* < 0.0001) [42]. A significant contribution of the minor allele T in the rs7903146 polymorphism of the *TCF7L2* gene to the development of T2D was observed in Spanish, Chinese, and Ghanaian populations [43,44,45,46]. Contradictory results exist, as a study conducted by Bahaaeldin, A.M. et al. did not confirm a significant association between the *TCF7L2* rs7903146 polymorphism and T2D [47]. Further research with larger sample sizes is needed. The obtained results align with studies conducted on Arabs in Saudi Arabia [48], the United Arab Emirates [49], Cameroon [50], Iran [51], and China [52], which also did not confirm a significant association between the rs7903146 polymorphism of the *TCF7L2* gene and the risk of developing T2D. The observed heterogeneity in studies of the rs7903146 polymorphism can be attributed to ethnic and racial differences in the studied populations. Smaller variations were found within Asian populations, while significant heterogeneity was detected among Europeans [53,54]. The genetic contribution of the *TCF7L2* rs7903146 polymorphism to the development of prediabetes has not been previously studied, which justifies the selection of this polymorphism for conducting replicative genotyping in cases of prediabetes within the Kazakh population. While our research specifically focuses on the Kazakh population, the findings may hold broader implications for comprehending the propensity to prediabetes among diverse ethnic groups. The genetic polymorphisms of *TCF7L2* and *PPARG*, explored in our investigation, assume global relevance due to the increasing prevalence of prediabetes and T2D on a global scale. 

Thus, the significant genetic contribution to the development of T2D, substantial ethnic differentiation, contradictory results of previous studies in assessing the strength of associations between the rs7903146 polymorphism of the *TCF7L2* gene and the risk of T2D, and the absence of similar studies on prediabetes, indicate the need for replicative associative studies of this polymorphism in cases of prediabetes within the Kazakh population. The distribution of genotypes for the two investigated polymorphisms, *TCF7L2* (rs7903146) and *PPARG* (rs1801282), in the Kazakh sample, as determined through statistical analysis using the PLINK-HWE test program, is consistent with Hardy–Weinberg equilibrium (*p* > 0.05).

A comparative analysis of genomic information from the Miras DNA bank with previously studied populations worldwide indicates a high genetic heterogeneity of the investigated polymorphisms, reflecting the population structure peculiarities of the Kazakh people, which have been shaped by complex evolutionary and migratory processes, as well as their intermediate geographic position between populations of East Asia, South Asia, and Europe. The comparative analysis of population frequencies of unfavorable alleles and genotypes of *TCF7L2* (rs7903146) and *PPARG* (rs1801282) confirms their high genetic heterogeneity, reflecting the population structure peculiarities of the Kazakh people and indicating the need for association genomic studies of prediabetes and T2D in each ethnic population. The early updated recommendations and studies over the world suggests the continuing of efforts to investigate the urgency and risks of prediabetes, diabetes, and its complications [55,56,57,58].

This study has a limitation due to the absence of a prediabetes or diabetes population and/or prospective follow-up. However, it is the first study in the Kazakh population aimed at determining the frequency of polymorphic variants in the *TCF7L2* (rs7903146) and *PPARG* (rs1801282) genes in healthy individuals.

## 5. Conclusions

The analysis of population characteristics of the distribution of allele and genotype frequencies of polymorphic genetic variants in the *TCF7L2* (rs7903146) and *PPARG* (rs1801282) genes revealed notable findings in the Kazakh population. Specifically, the minor G allele of the “Asian” protective polymorphism rs1801282 in the *PPARG* gene was observed at the highest frequency (13.8%) in the Kazakh population compared to European and other Asian populations studied. This finding suggests a more significant protective effect of this polymorphism in reducing the risk of prediabetes and type 2 diabetes in Kazakhs. The intermediate population frequency of the unfavorable T allele of the insulin secretion-disrupting gene *TCF7L2* (rs7903146) in Kazakhs (15.2%), compared to European and Asian populations, indicates that these genetic loci are not universally applicable genetic markers for a predisposition to T2D and should be replicated in each specific ethnic population. Further results from replicative genomic research will help identify significant polymorphic variants of genes underlying the alteration of prediabetes status.

## Figures and Tables

**Table 1:** Genetic characteristics of *PPARG* (rs1801282) and *TCF7L2* (rs7903146) gene polymorphisms associated with prediabetes according to GWAS.

No.	Gene Name	Chromosome	rs	Position
1	*PPARG*	3	rs1801282	12351626
2	*TCF7L2*	10	rs7903146	112998590

**Table 2:** Minor allele frequencies of the gene polymorphisms, *PPARG* (rs1801282) and *TCF7L2* (rs7903146), associated with the development of prediabetes, in the Kazakh population.

Gene Name	rs	MAF	*N*	A1	A2	GENO	Conformity of Genotype Distributions to Hardy–Weinberg Equilibrium		
							O (HET)	E (HET)	*p*
*PPARG*	rs1801282	0.1378	1800	C	G	1338/428/34	0.2378	0.2376	1
*TCF7L2*	rs7903146	0.1521	1801	C	T	1298/458/45	0.2543	0.258	0.5233

Footnotes: rs—identifier of the polymorphism (SNP Identifier); MAF—minor allele frequency; *N*—number of genotyped individuals; A1—wild-type allele and A2—minor allele; GENO—number of detected genotypes; O (HET)—expected heterozygosity according to Hardy–Weinberg equilibrium; E (HET)—observed heterozygosity according to Hardy–Weinberg equilibrium; *p*—observed significance of differences.

**Table 3:** Comparative analysis of allele frequencies of the *PPARG* (rs1801282) and *TCF7L2* (rs7903146) gene polymorphisms, GWAS-associated with prediabetes, in global populations.

Population	*N*	MAF	χ^2^	*p*
*PPARG* rs1801282				
Kazakhstan	1800	0.1378		
Europe	503	0.120	1.73	0.189
East Asia	504	0.026	92.02 *	<0.001
South Asia	489	0.120	1.828	0.177
England	91	0.121	0.209	0.648
Spain	107	0.117	0.227	0.635
Italy	107	0.084	2.494	0.115
China	103	0.049	6.730 *	0.010
Japan	104	0.029	10.194 *	0.002
Vietnam	99	0.01	13.427 *	<0.001
Bangladesh	86	0.11	0.765	0.382
India	103	0.092	2.119	0.146
Pakistan	96	0.141	0.050	0.824
*TCF7L2* rs7903146				
Kazakhstan	1801	0.1521		
Europe	503	0.317	105.41	<0.001
East Asia	504	0.023	114.31	<0.001
South Asia	489	0.299	83.79	<0.001
England	91	0.258	6.626 *	0.011
Spain	107	0.397	42.234 *	<0.001
Italy	107	0.374	36.105 *	<0.001
China	103	0.024	13.846 *	<0.001
Japan	104	0.029	12.027 *	<0.001
Vietnam	99	0.01	15.294 *	<0.001
Bangladesh	86	0.279	9.945 *	0.002
India	103	0.282	12.194 *	<0.001
Pakistan	96	0.25	6.592 *	0.011

Footnotes: *N* is the number of DNA samples; MAF is the frequency of the minor allele; χ^2^ is the Chi–square criterion; *p* is statistical significance; *—differences are statistically significant (*p* < 0.05), MAF data are presented from Ensembl genome database project—Ensembl (2021); http://asia.ensembl.org/index.html (accessed on 10 May 2024).

## Data Availability

The data are available upon request.
